# Retrospective Analysis of Clinical Performance of an Estonian Speech Recognition System for Radiology: Effects of Different Acoustic and Language Models

**DOI:** 10.1007/s10278-018-0085-8

**Published:** 2018-04-30

**Authors:** A. Paats, T. Alumäe, E. Meister, I. Fridolin

**Affiliations:** 1 Department of Health Technologies, Tallinn University of Technology, Ehitajate tee 5, 19086 Tallinn, Estonia; 2 Medical Technology Division, North Estonia Medical Centre, J. Sütiste tee 19, 13419 Tallinn, Estonia; 3 School of Information Technologies, Tallinn University of Technology, Ehitajate tee 5, 19086 Tallinn, Estonia

**Keywords:** Automatic speech recognition, Radiology, Estonian language, Spontaneous dictation, Word error rate

## Abstract

The aim of this study was to analyze retrospectively the influence of different acoustic and language models in order to determine the most important effects to the clinical performance of an Estonian language-based non-commercial radiology-oriented automatic speech recognition (ASR) system. An ASR system was developed for Estonian language in radiology domain by utilizing open-source software components (Kaldi toolkit, Thrax). The ASR system was trained with the real radiology text reports and dictations collected during development phases. The final version of the ASR system was tested by 11 radiologists who dictated 219 reports in total, in spontaneous manner in a real clinical environment. The audio files collected in the final phase were used to measure the performance of different versions of the ASR system retrospectively. ASR system versions were evaluated by word error rate (WER) for each speaker and modality and by WER difference for the first and the last version of the ASR system. Total average WER for the final version throughout all material was improved from 18.4% of the first version (v1) to 5.8% of the last (v8) version which corresponds to relative improvement of 68.5%. WER improvement was strongly related to modality and radiologist. In summary, the performance of the final ASR system version was close to optimal, delivering similar results to all modalities and being independent on user, the complexity of the radiology reports, user experience, and speech characteristics.

## Introduction

In the modern healthcare system, computers and electronic healthcare records are used extensively. Radiology is the most computerized specialty and a pioneer among other clinical fields using diagnostic workstations for image interpretation and radiology information systems for documenting findings. Demographic changes, including population aging, increase the demand for healthcare services. This trend has also influenced radiology, where, for example, in Estonia, the number of radiology procedures, carried out between 2010 and 2015, has increased by 30%. At the same time, the number of radiologists has remained similar (190 in 2007, 188 in 2015) [1]. This demonstrates clearly the deficit of well-qualified radiologists in Estonian healthcare sector.

To be able to fulfill all patient needs with limited resources, there is a necessity for radiologists to find a way for more effective image reporting. Currently, in Estonia, radiologists manually type results of visual findings and quantitative measurements of a study as a textual report. However, automatic speech recognition (ASR) has shown to be a valid alternative, replacing the traditional keyboard-based text entry in radiology reporting which can improve patient care and resource management in the form of reduced report turnaround times, reduced staffing needs, and the efficient completion and distribution of reports [2, 3].

Reporting by ASR is widely in use in countries where software solutions for local languages are available. ASR technology has been commercially available for languages with a large number of speakers (like English, French, German) already for several decades [3]. Estonia is a small multinational country with about 1.4 million inhabitants, among those 70% speaking Estonian and 30% other languages, mainly Russian. Native language-supported ASR systems for under-resourced and agglutinative languages are often not available [4, 5] which is also the case of Estonian language. Apart from a preliminary attempt [6] and the system presented in this paper, no Estonian-based ASR systems exist currently in radiology.

The scientists from Tallinn University of Technology (TTÜ), in collaboration with radiologists from North Estonia Medical Centre (NEMC), Tallinn, Estonia, took a step closer towards an ASR application in radiology for Estonian language by implementing an ASR prototype for Estonian language in radiology domain [7]. Due to the lack of resources and available commercial ASR system for Estonian language, open-source software components were utilized. Since ASR technology in its development phase has high frequency of transcription errors, necessitating careful proofreading and report editing, a profound understanding of the errors and the frequency of errors is inevitable. Effective utilization of the ASR system could be hampered by high error rate [8, 9], low acceptance, and interest by the radiologists due to the issues related to the workflow or culture [10, 11]. In order to achieve similar performance to the commercial systems in general, the first ASR system prototype developed for Estonian language in radiology domain was modified and improved further, including the integration of domain-adapted deep neural network (DNN)-based acoustic models (AM), language model (LM) adaptation using real dictated texts, smarter handling of sentence breaks, and spoken noises in the language model [12]. As a prerequisite for successful exploitation, modified software demand tests in clinical environment to reveal the dictation error rates in finalized radiology reports.

The aim of this study was to analyze retrospectively the influence of different acoustic and language models in order to determine the most important effects to the clinical performance of an Estonian language-based non-commercial radiology-oriented automatic speech recognition (ASR) system.

## Materials and Methods

The ASR system was utilizing free- and open-source software (Kaldi toolkit,^1^ Thrax^2^) [7, 12] based on server-client platform developed in TTÜ^3^^,^^4^. System components in server side, responsible for converting dictated speech into text, were available for clients over network as reported earlier [13]. Client side system component, responsible for collecting the audio, converting it into digital, and sending it to the server for processing, receiving and representing recognized text, was implemented as a Java application and was available for radiologists as a web-based tool.

ASR system was traditional, using an AM and a LM for speech-to-text transformation [3]. Source textual information necessary for preparation of the text corpus were collected and prepared in NEMC based on real radiology reports. Normalization of text corpus and training of LM was done as described earlier [7, 12]. During the development, feedback was collected from daily ASR system users [7] and the ASR system characteristics were modified in order to minimize errors through the enhancement of the AM or LM (Table 1) reported in detail earlier [12]. In the first version of the ASR system, the Gaussian mixture model (GMM)-based acoustic model was used as described elsewhere [7]. During later enhancements, the AM was improved to include the integration of DNN technology [12].

**Table 1:** Different ASR system development versions

Version number	ASR system characteristics
v1	GMM acoustic model, language model trained on 1-year reports
v2	DNN acoustic model, language model trained on 1-year reports
v3	+ language model trained on 5-year reports
v4	+ better noise modeling in language model
v5	+ better modeling of sentence breaks
v6	+ less aggressive silence detection
v7	+ acoustic model adapted using in-domain data
v8	+ language model adapted using spoken data

The final version of the ASR system (v8) and the web-based tool was used during routine reporting process in clinical practice at the NEMC radiology department by 11 radiologists. This collected dataset was used to estimate retrospectively the performance of each ASR system version to evaluate every model version in the similar conditions. Also, this avoided the learning bias being built up in different development phases of the ASR system.

Radiologist’s standard workplace consists of a PC equipped with four monitors. One monitor was used for composing a report in the Radiology Information System (RIS) and the others for visualization of images with PACS (Picture Archiving and Communication System) client (Agfa, Impax 6.4). A web interface of the ASR prototype was implemented into the same monitor as RIS in a way that the radiologist had visual control of both systems at the same time. Every station where prototype was tested was equipped with a high-quality microphone headset (Logitech USB H340).

Radiologists were supplied with written instructions of experiments that explained how to select reports, connect and adjust microphone, start ASR client software, and spell punctuations and abbreviations. Dictations were marked with a unique code and stored by the web application, allowing to identify every individual study and the modality during analyzing process. Additional information about the radiologist carrying out the dictation process was included.

Every radiologist reported approximately 20 radiological studies in spontaneous dictation manner. For guaranteeing uniform distribution of report types, there was a recommendation to report eight computed tomography (CT), four magnet resonance tomography (MR), four X-ray (XR), and four ultrasound (US) studies. The radiologists, specialized in certain modalities (e.g., CT, MR), reported only those modalities and did not report other modalities. Distribution of dictated reports and modalities between radiologists is presented in Table 2. Average number of words per report for each modality was 126 (SD 66.6) for CT; 90.0 (SD 33.5) for MR; 37.2 (SD 21.7) for XR; and 74.6 (SD 42.3) for US. The total number of words for each modality was CT 11083, MR 3778, XR 1561, and US 3506.

**Table 2:** Distribution of dictated reports among radiologists as “Total no. reports” and modalities (XR X-ray, CT computed tomography, MR magnetic resonance tomography, US ultrasound). The number of total words per radiologist is given as “Total no. words“

Radiologist	Total no. reports	Modality				Total no. words
		CT	MR	XR	US	
No. 1	19	8	3	4	4	2006
No. 2	19	7	4	4	4	1250
No. 3	22	9	13			2031
No. 4	22	10	4	4	4	2463
No. 5	19	8		9	2	1675
No. 6	20	8	10	2		1875
No. 7	20	8	8		4	2057
No. 8	20	8		4	8	1693
No. 9	19	6			13	1701
No. 10	19	8		7	4	1409
No. 11	20	8		8	4	1768
Total	219	88	42	42	47	19,928
Mean	19.9	8.0	7.0	5.3	5.2	1811
SD	1.1	1.0	4.0	2.4	3.3	331

The dictations recorded during the testing were analyzed. For this purpose, every dictated audio file was carefully listened and the content was transcribed into a text file as it was spoken. Every text file was used as a reference for comparing output text produced by the ASR system from dictated audio.

The comparison revealed a difference between reference and recognition resulting in a number of incorrectly recognized words characterized with a number of substitutions (S), number of deletions (D), number of insertions (I), and correct words (C) in the synthesized text. Those variables were used to calculate word error rate (WER) for each dictated report with every ASR system model version as:

1 $$ \mathrm{WER}=\frac{S+D+I}{N}=\frac{S+D+I}{S+D+C} $$

where *N* is a number of words in the reference texts [3, 14, 15].

The performance improvement of the last edition of the ASR system (v8), compared to the first one (v1), was characterized by the WER difference between the systems:

2 $$ {\mathrm{WER}}_{\mathrm{difference}}={\mathrm{WER}}_{\mathrm{v}1}-{\mathrm{WER}}_{\mathrm{v}8} $$

where WER_v1_ and WER_v8_ are the WER values calculated for the model versions v1 and v8, respectively.

The WER and the WER difference values for each dictation were collected into a database to evaluate recognition accuracy of each ASR system version, modality, and radiologist. Total WER, WER by radiologist, and WER by modality were calculated and presented as mean percentage value together with standard deviation (SD) for each ASR system version. Additionally, WER difference by radiologist and modality were determined and exhibited as median, maximum, minimum, first, and third quartile. The paired Student’s *t*-test for means was applied to compare means for WER, and the resulting *p* < 0.05 was considered significant.

## Results

**Fig. 1:**
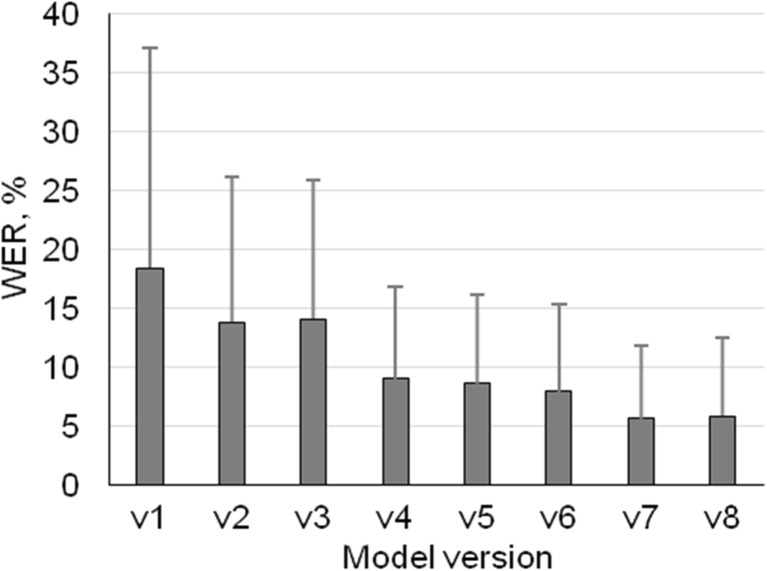
The total WER (mean, SD) for model versions 1 to 8

Figure 1 demonstrates changes in total WER over all dictated reports for all system versions. In the first version (v1) of the ASR system with GMM acoustic model and language model trained on 1-year reports, the total WER was 18.4% (SD 18.6). Next, version (v2) of ASR system with DNN acoustic model and a language model trained on 1-year reports reduced total WER significantly to 13.8% (SD 12.4, *p* < 0.05). Interestingly, using the language model trained on 5-year reports, the ASR version v3 did not lower total WER compared to v2, but better noise modeling in the language model decreased WER from 14.1% (SD 11.8) to 9.0% (SD 7.8, *p* < 0.05) in the case of v4. Enhanced modeling of sentence breaks in the ASR system (v5) and less aggressive silence detection (v6) did not generate large difference compared to v4, since the total WER decreased only to 7.9% (SD 7.4), but were still statistically significant (*p* < 0.05). The ASR system version v7 that incorporated speaker-specific acoustic models adapted to audio files reduced errors even more, delivering a WER of 5.6% (SD 6.2, *p* < 0.05). Adapting the language model with previously dictated texts (v8) did not have a big impact. WER stayed almost the same with a small increase to 5.8% (SD 6.6, *p* = 0.177). Large SD values are indicating heterogeneity of individual dictations and substantial differences in recognition accuracy.

**Fig. 2:**
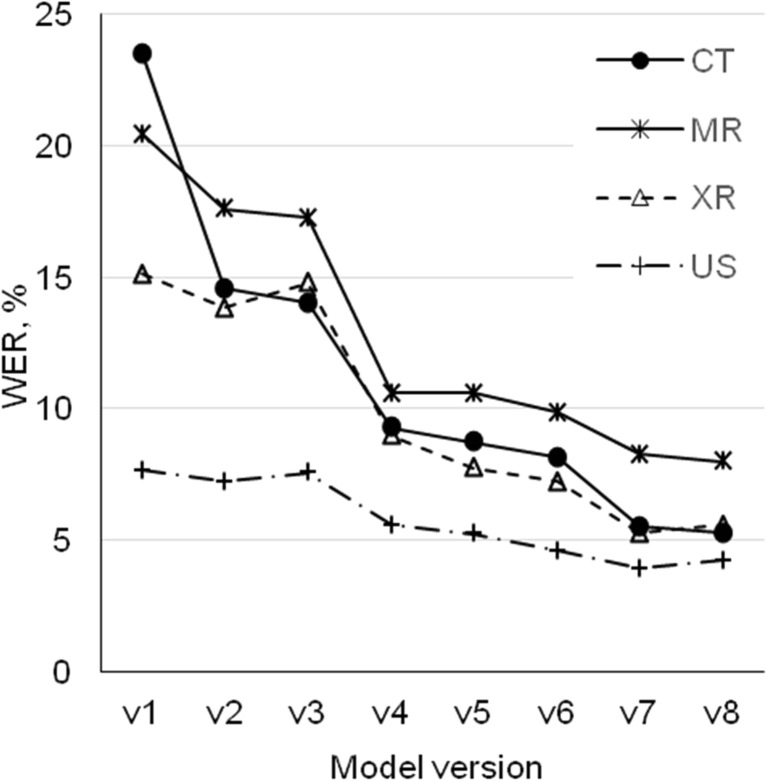
Word error rates by modality for different model versions

The WER data by modality for each system version exhibit generally similar decreasing trend except for US, which has low WER already for v1 (Fig. 2). WER was improved throughout all model versions for different imaging modalities. The system accuracy of the first system version was different for every modality, starting with mean WER 23.5% (SD 21.9) for CT and 7.6% (SD 7.1) for US, and achieving the WER value of 5.3% (SD 4.8) for CT and 4.9% (SD 5.1) for US in the final system version (*p* < 0.05).

**Fig. 3:**
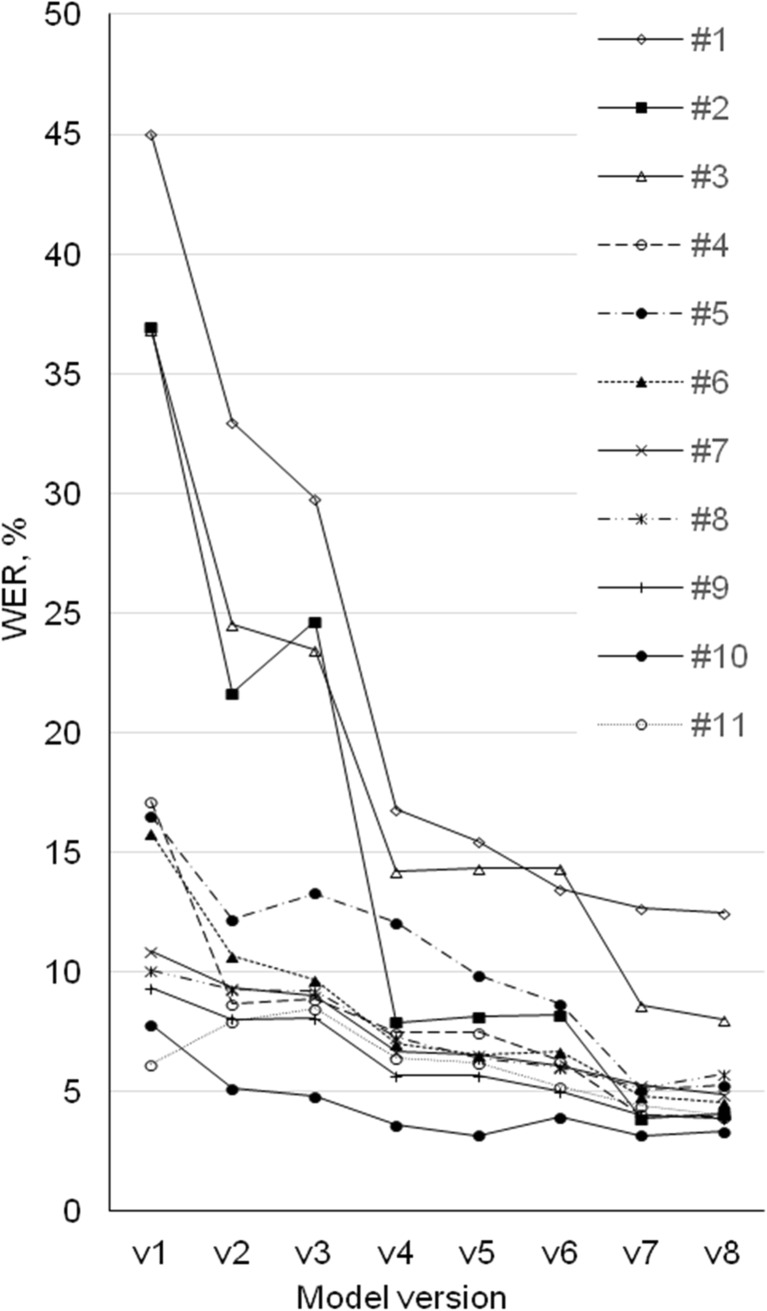
Word error rates corresponding to individual radiologist for different model versions

Figure 3 shows the word error rates corresponding to individual radiologist for different system versions. A common trend for each radiologist is similar: the last system model version is giving generally better performance than the first. However, the difference in outcome of the system for an individual radiologist is clearly visible. Higher WER values for radiologists no. 1, no. 2, and no. 3 compared to others are seen. Moreover, the declining trend of WER is discontinued for some middle system versions where the number of errors for some radiologist (e.g., for radiologist no. 2, no. 4, no. 5, no. 9, no. 11) increased.

**Fig. 4:**
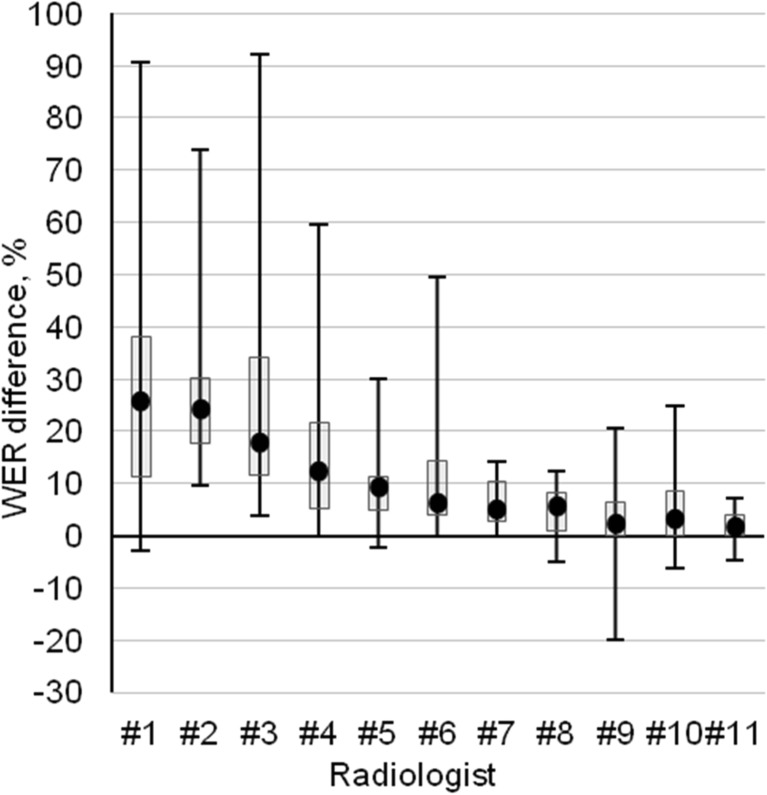
Median word error rate improvement with maximum, minimum, first, and third quartile between the first (v1) and the last (v8) model versions corresponding to individual radiologist

**Fig. 5:**
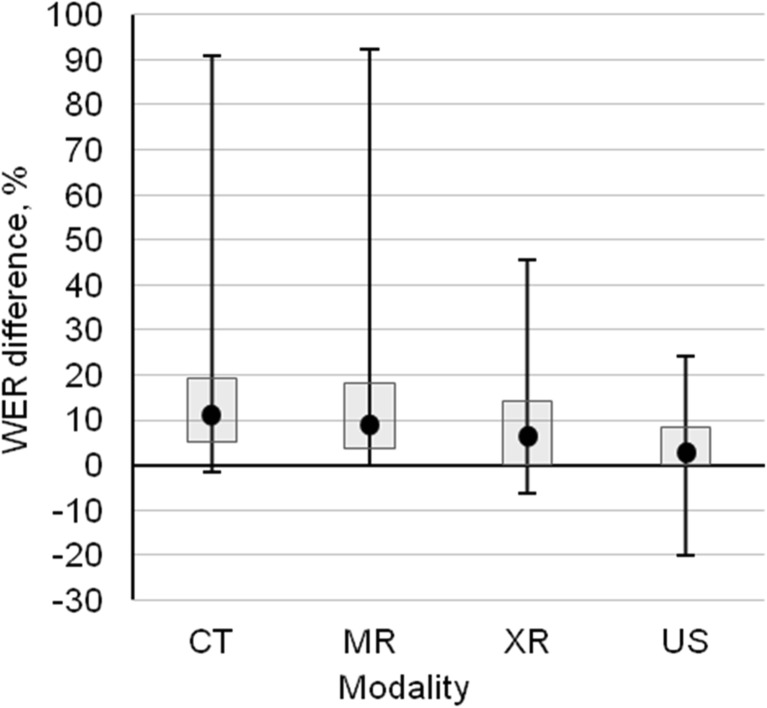
Median of word error rate improvement with maximum, minimum, first and third quartile between first (v1) and last (v8) model versions by modality

The high value of standard deviation made it problematic to evaluate the recognition improvement between the first (v1) and final (v8) ASR system models by mean and SD. For this reason, the results in Figs. 4 and 5 are presented as median, quartiles, and minimum and maximum values.

Improvement of the system as a difference of WER between the first (v1) and last (v8) system versions for each radiologist is presented in Fig. 4. For most of the radiologists, the ASR system’s performance improved with the system version v8, compared to that of the system version v1. However, the improvement rate for individual radiologists was different. In the best case (radiologist no. 3), the max WER improved by 91%. The median WER difference ranged from 25.6% for radiologist no. 1 to 1.6% for radiologist no. 11.

Figure 5 studies the model improvement for each modality, presenting the highest score for CT (median WER difference 11.8%) and lowest for US (median WER difference 2.6%) between the first and the last versions. The ASR system version v8 gave higher recognition accuracy (WER difference > 0) compared to the version v1 in 179 dictations from the total amount of 219. Only 16 dictations (CT 2/88; XR 4/42, US 10/47) had better result with ASR system version v1 than with version v8 (WER difference < 0). On 24 cases (CT 4/88; MR 2/42; XR 8/42; US 10/47), the recognition accuracy was the same for versions v1 and v8. Almost all dictated reports benefited from the final system version for CT and MR. Despite the system improvement being the smallest for US, the 82% of the dictated reports achieved better outcome in the final system version, compared to the first.

## Discussion

The ASR system architecture utilized in our approach is similar to the other ASR applications for radiology [13, 16].

The data on Fig. 1 shows that the system version v1 had similar WER as other GMM-based models (18.41%) used for radiology ASR systems reported by Miranda et al. 2008 [13]. When increasing the number of training reports in the ASR system’s language model from 1-year data (v2) to 5-year data (v3), the changes were negligible (*p* = 0.239). Even a small gain of error was detected as WER increased from 13.8% (SD 12.4) to 14.1% (SD 11.8) indicating that system version v1 had sufficiently diverse dataset based on 1-year radiology reports for language model training. Adding the dataset based on 5-year reports did not improve the performance.

Comparing our open-source software-based ASR system performance to commercially available products, the overall error rate is in the same order or even lower. For example, the overall error rate in the study by the IBM MedSpeak ASR system was found to be 10.3% (SD 3.3) [17]. Using the ASR system Nuance Gen, Nuance Med, and SRI Decipher for interpreting spoken clinical questions resulted in a WER of 68.1, 67.4, and 26.7%, respectively [18]. After all model modifications, our radiology domain-specific system performance improved from 18.4% to a final WER of 5.8%, which yields relative WER improvement of 68.5%. This behavior is similar to that of the SRI system improvement of 36% applied to general clinical text [18]. The SD value of WER (18.7 and 6.6% for system version v1 and v8, respectively) for our ASR system was somewhat higher than that in earlier studies [17], probably explained by more heterogeneous report set in our study.

Figures 2 and 3 reveal the impact of different system versions to the imaging modalities and individual radiologists. The implementation of the ASR system version v2 reduced WER for all modalities and all radiologists except for radiologist no. 11 (13 reports from 20 had higher WER in v2 than in v1). The possible cause was that GMM acoustic model in v1 fitted well with the voice characteristics of the radiologist no. 11, which was lost in the model v2 with a more general DNN acoustic model.

The ASR system version v3 had a small effect on recognition errors being not statistically relevant for any of modalities (*p* > 0.05). WER drifted bi-directionally up and down (Figs. 2 and 3) and for some radiologists (radiologist no. 2 and no. 5), performance worsened (*p* < 0.05). This could be explained by a large number of rarely occurring words in language model dictionary based on the dataset of 5-year reports. In the reports for modalities like US and XR with relatively simple vocabulary, many additional alternatives created a situation where probability to find correct word was more complex.

Implementation of the ASR system version v4 affected all radiologists, except no. 4, and modalities in a similar way, the improvement of WER was significant (*p* < 0.05). This can be explained by the reduction of filler non-stationary noises and enhanced background noise processing (e.g., elimination of sounds coming from keyboard, mouse, etc).

The ASR system versions v5 and v6 reduced WER in a small scale for all modalities, but influence to the radiologist dictations was different. For some radiologists, WER decreased and for others, it increased, because those models attempted to simulate specific work situations (e.g., long pauses caused by performing measurements on image during dictation, a pause of thought) where users had different behaviors.

Additional progress was detected after implementation of the ASR system version v7 as WER decreased for all radiologists and modalities, except for radiologists no. 1, no. 8, and no. 11 and for US modality (*p* > 0.05). This can be explained with better performance of the acoustic model tuned with audio files dictated by radiologists from previous tests.

The ASR system v8 had a relatively small, non-significant (*p* > 0.05) impact: WER decreased for CT and MR but increased slightly for XR, for US, and for some radiologists (no. 2, no. 5, no. 8, no. 10). By adapting the language model, including content from earlier dictated reports, WER improved for more complicated CT and MR reports, but not for more standardized US and XR reports.

The results in Figs. 4 and 5 characterize relative improvement in recognition accuracy for individual radiologists and modalities between the first and last versions of the ASR system as WER difference. The ASR system v8 displayed significant improvement in recognition accuracy to the most of the CT and MR report dictations, compared to the system version v1. For the XR and US reports, the improvement was smaller, but enough to guarantee as high or even better detection accuracy than for CT and MR, referring to relatively good recognition algorithms already used in the ASR system version v1 for XR and US. Generally, the ASR system provided the lowest WER for US, in comparison to other modalities. It can be explained by US reports having a more standardized structure than others. For MR, the ASR did not reach the same performance level as for XR and CT (Fig. 2). Similar to our results, Ramaswamy et al. [19] used the ASR system for dictation of MR reports and achieved an average WER of 7.3%. Another study investigated CT and MR reports and indicated an average WER of 2.81% [11] and between 7.8–11.5% and 9.3–10.6% for CT and MR, respectively [17].

Changes in different ASR system versions were made to increase low recognition accuracy, mostly induced by the reports of complicated 3D modalities (CT and MR). This task is fulfilled for all ASR system versions. At the same time, modalities already having a good recognition accuracy (XR, US) tend to suffer from this and WER increased slightly in some system versions compared to the previous ones. The reason lies probably in a large number of rarely occurring words in language model dictionary based on the dataset of 5-year reports as explained above.

It is important that for the final version of the ASR system, the accuracy is similar for all modalities and WER is in the range of 4.2–8.0%. However, there is no practical need to implement technology used in the ASR system version v8 for US and XR modalities since the model v7 assures the same result. According to the IBM Watson team [20], human accuracy as WER in English conversational speech recognition was reported around 5.1% and has been estimated to be even as low as 4% [21]. Our free- and open-source software-based ASR system approaches this number although the human transcription accuracy for dictated radiology reports is probably much lower than for conversational telephone calls. Moreover, an exact comparison is difficult, because WER values reported by different authors vary, probably caused by differences in methodology, study group, the complexity of reported studies, etc.

In summary, the performance of the final ASR system version was close to optimal, delivering similar results to all modalities and being independent on the user, the complexity of the radiology reports, user experience, and speech characteristics. Even if some ASR system model versions did not give statistically significant improvements, they cannot be ignored and should be considered for implementation due to the fact that the effect was present for some radiologists.

## Conclusions

This study contributes to the knowledge how different characteristics of the acoustic and language models of the ASR system based on open-source software can improve ASR system performance in radiology domain for a small language as Estonian. Hopefully, this preserves native language-based working environment in clinics under the pressure of fast-developing technology and globalization.

## Footnotes

1
https://github.com/kaldi-asr/kaldi

2
http://www.openfst.org/twiki/bin/view/GRM/Thrax

3
https://github.com/alumae/kaldi-gstreamer-server

4
https://github.com/alumae/gst-kaldi-nnet2-online
